# Association of the energy-adjusted dietary inflammatory index and Sjögren’s syndrome: a cross-sectional study

**DOI:** 10.1017/S0007114525103474

**Published:** 2025-06-14

**Authors:** Ezgi Karataş, Fatih Taştekin, Figen Yargucu Zihni, Burcu Barutçuoğlu, Gonca Karabulut

**Affiliations:** 1 Ege University, Faculty of Health Sciences Nutrition and Dietetics Department Suat Cemile Balcıoğlu Campus Karşıyaka, İzmir, Turkey; 2 Ege University, Faculty of Medicine, Department of Rheumatology Bornova Campus, İzmir, Turkey; 3 Ege University, Faculty of Medicine, Department of Medical Biochemistry Bornova Campus, İzmir, Turkey

**Keywords:** Sjogren’s syndrome, Dietary Inflammatory Index, Nutrition assessment, Inflammation

## Abstract

There are no studies in the literature examining the inflammatory content and effects of the diets of patients with primary Sjögren’s syndrome (PSS). This study aimed to investigate the relationship between the Energy-Adjusted Dietary Inflammatory Index (E-DII) and anthropometric measurements, disease activity, inflammatory markers, and blood lipid concentrations in female patients with PSS. A cross-sectional study was conducted between November 2020 and November 2021, including 102 female patients with a confirmed diagnosis of PSS. Dietary intake was assessed using the multiple-pass, 3-day food record method to calculate E-DII scores. Physical activity was evaluated using the International Physical Activity Questionnaire (Short Form), anthropometric measurements were taken, and the European League Against Rheumatism Sjögren’s Syndrome Disease Activity Index (ESSDAI) was used to determine disease activity. Lipid profile and inflammatory markers were analyzed in blood samples. Patients had a median E-DII value of –2·36. An anti-inflammatory diet was associated with lower anthropometric measurements and reduced total cholesterol, triglycerides, and low-density lipoprotein cholesterol concentrations. Logistic regression analysis revealed a significant association between E-DII and complement C3 (OR: 1·03, 95 % CI: 1·00, 1·05, *P* = 0·021) and C4 (OR: 1·08, 95 % CI: 1·01, 1·13, *P* = 0·019) after adjusting for age, disease score, drug use for SS, smoking, and physical activity. However, no significant correlation was found between E-DII and ESSDAI scores, C-reactive protein, or erythrocyte sedimentation rate. In conclusion, a pro-inflammatory diet was associated with higher anthropometric measurements and adverse lipid profiles in PSS patients, but its relationship with disease activity and inflammation remains unclear.

## Graphical abstract

**Figure:**
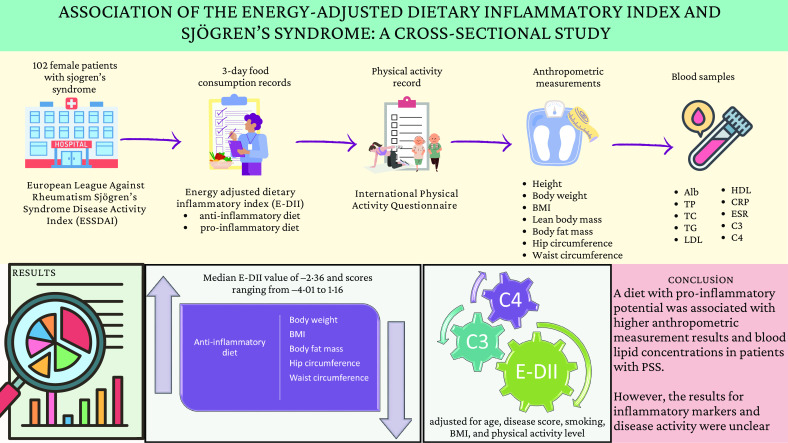

Sjögren’s syndrome (SS) is a chronic autoimmune disease affecting middle-aged women, with its prevalence varying between 0·01 % and 0·05 %^(1)^. Primary SS (PSS) is characterized by lymphocyte infiltration and destruction of salivary and lacrimal glands and extra-glandular manifestations in the lungs, kidneys, nervous system, skin, and musculoskeletal system^(2)^. Assessing disease activity is difficult in most patients because the predominant symptoms are relatively stable. The European League Against Rheumatism (EULAR) Sjögren’s Syndrome Disease Activity Index (ESSDAI) is used to assess disease activity^(3)^. Besides genetic and epigenetic factors, potential environmental risk factors, such as a history of autoimmune disease in first-degree relatives, infections, and adverse stressful living conditions, also affect the occurrence of PSS^(4)^. C-reactive protein (CRP) levels and erythrocyte sedimentation rate (ESR) are significantly higher in patients with PSS than in healthy individuals and correlate strongly with disease activity. No response to treatment is seen unless the levels of these markers of inflammation decrease^(5)^. Diet is a modifiable environmental factor that plays a crucial role in regulating inflammation and immune function^(6)^. The intake of foods with anti-inflammatory properties, such as fish, vitamin A, vitamin C was found to be associated with a lower risk of PSS^(7)^. Similarly, an animal model study investigating the effect of a gluten-free diet on salivary gland and pancreatic islet inflammation found that a gluten-free diet reduced the infiltration of monocytes/macrophages and T cells in salivary glands and inflammation in pancreatic islets^(8)^. In nutrition research, several diet quality indices have been designed to assess the effects of inflammation and overall diet. Among these, the most widely used Dietary Inflammatory Index (DII) is designed to measure the potential inflammatory properties of a diet^(9)^. Some previous studies have examined the association between DII and several clinical conditions, including cardiovascular disease and diabetes^(10,11)^. Recent clinical studies have demonstrated an association between DII and a rheumatologic autoimmune disease such as PSS^(12–14)^. Considering that diet may be effective in inflammatory processes and the inflammatory potential of diet in patients with PSS has not been investigated, this study was performed to investigate the relationship between DII score and anthropometric measurements, disease activity, inflammatory markers, and blood lipids in female patients with PSS.

## Materials and methods

A cross-sectional study was conducted between November 2020 and November 2021 involving 102 female patients aged 19 years or older and meeting the inclusion criteria. These patients were admitted to the Ege University Department of Internal Medicine, Division of Rheumatology, and had a confirmed diagnosis of PSS by a specialist doctor. After collecting patient anamnesis and examining their medical records, detailed information about the study was provided to the patients, and informed consent was obtained.

### Population and sample of the study

All female patients diagnosed with PSS who applied to the outpatient clinic of the Ege University Faculty of Medicine, Department of Internal Medicine, Division of Rheumatology, were invited to participate in the study. This study was approved by the Medical Research Ethics Committee of Ege University Faculty of Medicine. No: 21-10.1T/27. According to the results of G * Power analysis, 119 people were included in the study with 95 % power (1-*β*); moderate effect size (ρ:0·15); 0·05 sampling error (*α*)^(13)^. A total of 119 female patients who agreed to participate and met the inclusion criteria were included in the study. During the study, one patient died, two patients were diagnosed with cancer, the biochemical findings of four patients could not be completed, four patients wanted to leave the study, and the food consumption records of six patients could not be completed.

The inclusion criteria included the following: female patients aged 19 years and older, diagnosis of PSS by a rheumatologist using the American-European criteria^(15)^, and clinical stability with no change in medical treatment for 6 months prior to the study. The exclusion criteria included the following: pregnant or breastfeeding mothers, presence of any mental disease (schizophrenia and dementia), eating disorders (anorexia and bulimia), cancer, chronic renal failure, and chronic liver failure.

### Data collection

A questionnaire form prepared to determine the sociodemographic characteristics, medical history, and dietary habits of the patients, The multiple-pass 3-day food consumption record form, and the International Physical Activity Questionnaire (Short) – IPAQ (short) was used to collect the data. Anthropometric measurements were taken, and the disease activity index (European League Against Rheumatism) Sjögren’s syndrome disease activity index (EULAR Sjögren’s Syndrome Disease Activity Index (ESSDAI) was applied to determine disease activity. Additionally, biochemical data were obtained.

### Food intake and DII scores

The food consumption records were obtained by a nutritionist for 3 consecutive days, including 2 weekdays and 1 weekend day, to evaluate the daily energy and nutrient intakes of patients with PSS participating in the study. The average daily macro- and micronutrient intakes were calculated using the ‘Computer Assisted Nutrition Program, Nutrition Information Systems Package Program’ (BeBIS 8·2) program developed for Turkey. An average of 3 d was taken. DII scores were calculated using the method described by Shivappa et al.^(9)^ for each food or nutrient individually, and these values were then summed to obtain the overall DII score for each individual. The calculation was based on the average daily intake values for 42 foods and nutrients. Foods and nutrients that were not included in the BeBIS program (flavan-3-ol, flavones, flavonols, flavonones, anthocyanidins, isoflavones, and eugenol) and whose consumption was extremely limited in Turkey (rosemary and saffron) were not used for calculating the DII score. Among the nutrients, the contents of green tea and black tea were calculated based on their dry weights. When calculating the DII score, the consumption amounts of onion, garlic, pepper, thyme, and ginger were manually determined by analyzing the total amount of ingredients in the dishes within the BeBIS program. Subsequently, the consumption values of the relevant nutrient parameters were calculated manually. All reported intakes of nutrient parameters were converted into the amount per 1000 kcal energy intake to calculate energy-DII (E-DII) scores^(16)^. The lowest and highest values of the E-DII score were – 4·01 and 1·16, respectively. After the E-DII calculation, the participants were divided into two groups based on their E-DII scores from the median value. A diet with an E-DII score < –2·36 was classified as anti-inflammatory, and a diet with a score > –2·36 was categorized as pro-inflammatory.

### Anthropometric measurements

Participants’ height (cm), body weight (kg), waist circumference (cm), and hip circumference (cm) were measured by the researcher, and their body mass index (BMI) was calculated. The body composition of the participants was evaluated using the Tanita BC-418 MA device by the bioelectrical impedance analysis method. The body fat mass (kg) and lean body mass (kg) were analyzed based on the differences in electrical conductivity of adipose tissue and lean tissue mass^(17)^.

### European league against rheumatism Sjögren’s syndrome disease activity index (ESSDAI)

The ESSDAI, developed by a consensus of researchers from Europe and North America and supported by EULAR, was used to assess disease activity in patients with PSS. ESSDAI comprises 12 components, including evaluations of skin, respiratory, renal, joint, muscular, peripheral nervous system, central nervous system, hematologic, glandular, constitutional, lymphadenopathic, and biologic aspects. Each component is further divided into three to four activity levels, all accompanied by detailed descriptions^(18)^.

### Biochemical measurements

Total protein, albumin, triglycerides, total cholesterol, high-density lipoprotein (HDL) cholesterol, low-density lipoprotein (LDL) cholesterol, and CRP measurements were performed using the Cobas ® 8000 Modular analyser (Roche Diagnostics, Mannheim, Germany) in the Clinical Biochemistry Laboratory of Ege University Medical Faculty. Complement C3 (mg/dl) and complement C4 (mg/dl) measurements were performed using the Dade-Behring BN II Nephelometer (Siemens Healthineers, Germany). The ESR (mm) was determined using the modified Westerngren method on the Starrsed RS (Sysmex Europe GmbH) automatic ESH device.

### Statistical analysis

The data obtained from the study were analyzed using the IBM SPSS Statistics Standard Campus Edition package program 25·0. Descriptive statistics were expressed as mean (standard deviation) for variables with normal distribution and median [interquartile range (IQR)] for variables with non-normal distribution. Continuous variables were considered as numbers and percentages. The normality of the data sets was determined using the Shapiro–Wilk test, histograms, normality graphs, and kurtosis and skewness. The independent-samples *t* test was performed for normally distributed groups and the Mann–Whitney U test for non-normally distributed groups to examine the relationship between different variables and DII groups. Logistic regression was performed to determine the relationship between E-DII groups and anthropometric measurements, blood lipids, and inflammatory markers. The models were constructed with the enter method, and the analysis was adjusted for age, disease score, drug use for SS, physical activity level, smoking, and BMI. The overall goodness-of-fit of the models was assessed by Hosmer and Lemeshow test. The Omnibus test was used to analyze the significance of the models developed. The results of the logistic regression analysis were shown as *β*-coefficients [95 % confidence interval (CI)] and *P* values.

## Results

The sociodemographic and clinical characteristics of 102 female patients with PSS are presented in Table 1. When E-DII scores were divided into two groups based on the median value, the mean E-DII value was –2·89 (sd 0·45) and –1·46 (sd 0·65) in the anti-inflammatory (–4·01 to –2·36) and pro-inflammatory diet (–2·36 to 1·16) groups, respectively. According to the E-DII scores, no significant difference was observed in the sociodemographic characteristics of the patients, such as educational status, marital status, employment status, and social security.

**Table 1.** Characteristics of patients with Sjögren’s syndrome according to the E-DII score (Mean values and standard deviations; numbers and percentages; median values and interquartile ranges)

	Median E-DII ≤ –2·36 (*n* 51)		Median E-DII > −2·36 (*n* 51)		
	X	sd	X	sd	*P*
Age	57·39	9·99	52·88	13·8	0·058^†^
	*n*	%	*n*	%	
Marital status					
Married	39	76·5	36	70·6	0·529^‡^
Single	12	23·5	15	29·4	
Education level					
Primary school	25	49·0	15	29·4	
Middle school	5	9·8	6	11·8	0·133^‡^
High school	5	9·8	12	23·5	
University	16	31·4	18	35·3	
Employment status					
Full day	8	15·7	13	25·5	0·211^‡^
Part time	3	5·9	6	11·8	
Not working	40	78·4	32	62·7	
Social security					
Yes	45	88·2	47	91·2	0·741^‡^
No	6	11·8	4	7·8	
Smoking					
Yes	4	7·8	5	9·8	
No	44	86·3	43	84·3	0·941^‡^
Dropped out	3	5·9	3	5·9	
Drug use for SS					
Yes	27	52·9	32	62·7	0·211^‡^
No	24	47·1	19	37·3	
Types of drug used for SS*					
Hydroxychloroquine	26	60·5	37	71·2	
NSAID	2	4·7	4	7·7	0·984^‡^
Steroid	8	18·5	6	11·5	
Other DMARD	6	14·0	4	7·7	
Immunosuppressive	1	2·3	1	1·9	
	µ	IQR	µ	IQR	
Physical activity, min/week	925·00	891·00	1014·00	1747·00	0·616^§^
	*n*	%	*n*	%	
Physical activity level					
Inactive	26	51·0	29	56·9	
Minimal active	20	39·2	12	23·5	0·147^‡^
Very active	5	9·8	10	19·6	
	X	sd	X	sd	
Sjögren’s disease age	12·73	6·90	12·25	7·33	0·734^†^
E-DII score	–2·89	0·45	–1·46	0·65	
	µ	IQR	µ	IQR	
ESSDAI score	2·00	6·00	2·00	6·00	0·910^§^
	*n*	%	*n*	%	
ESSDAI classification					
Low	29	56·9	34	66·7	
Moderate	19	37·2	13	25·5	0·435^‡^
High	3	5·9	4	7·8	

DMARD = Disease-modifying antirheumatic drugs; E-DII = Energy-adjusted dietary inflammatory index; ESSDAI = European League Against Rheumatism (EULAR) Sjögren’s Syndrome Disease Activity Index; NSAID = Nonsteroidal anti-inflammatory drug; SS = Sjögren’s syndrome.

*There is more than one drug use.

Bold values indicate statistically significant differences (p < 0.05).

† Student *t* test mean (sd) deviation.

‡ Chi-square test.

§ Mann Whitney *U* median (IQR).

When assessing the medical status of the patients, no correlation was found between the E-DII scores and drug use or specific drug types. According to E-DII scores, the disease duration was 12·73 (sd 6·90) years and 12·25 (sd 7·33) years for those consuming the anti-inflammatory and pro-inflammatory diets, respectively. The inflammatory content of the diet was not associated with the disease score (ESSDAI). Similarly, no statistically significant relationship association was found in the ESSDAI classification between those who consumed anti-inflammatory diets and those who did not. The median IQR physical activity value of patients with PSS consuming anti-inflammatory and pro-inflammatory diets was 925·00 (891·00) min/week and 1014·00 (1747·00) min/week.

As depicted in Table 2, the E-DII scores of patients with PSS were categorized according to the median value, and the changes in anthropometric measurements and biochemical findings were evaluated. Patients consuming the anti-inflammatory diets had lower body weight, BMI, body fat mass, and hip and waist circumference measurements than those consuming the pro-inflammatory diets. Similarly, the blood concentrations of lipids other than HDL cholesterol were better in the group consuming the anti-inflammatory diets. Among the inflammatory markers, the levels of complements C3 and C4 were statistically higher in the group consuming the pro-inflammatory diets, whereas no significant difference was found in CRP levels and ESR.

**Table 2.** Distribution of anthropometric measurements and biochemical findings according to the E-DII score in patients with Sjögren’s syndrome (Mean values and standard deviations; median values and interquartile ranges)

	Dietary Inflammatory Index Score				
	Median E-DII ≤ –2·36 (*n* 51)		Median E-DII > −2·36 (*n* 51)		*P*
	Mean	sd	Mean	sd	
Anthropometric measurements					
Height (m)	1·58	0·07	1·57	0·06	0·306^*^
Body weight (kg)	68·0	10·5	74·16	12·9	**0·010** ^*^
BMI (kg/m^2^)	27·1	5·3	30·3	5·6	**0·004** ^*^
Lean body mass (kg)	48·5	9·4	48·4	6·6	0·956^*^
Body fat mass (kg)	29·5	8·1	34·1	8·1	**0·005** ^*^
Hip circumference (cm)	109·1	11·1	115·0	11·2	**0·010** ^*^
Waist circumference (cm)	95·6	14·3	97·5	11·7	**0·001** ^*^
Biochemical findings					
Albumin (g/l)	48·85	3·60	44·89	2·63	0·934^*^
Total protein (g/l)	73·73	5·60	73·69	6·40	0·972^*^
Total cholesterol (mg/dl)	187·76	34·21	202·88	38·47	**0·039** ^*^
TAG (g/dl)	102·08	43·37	126·10	54·19	**0·015** ^*^
LDL-cholesterol (mg/dl)	110·88	29·73	125·16	37·67	**0·036** ^*^
HDL-cholesterol (mg/dl)	61·33	23·16	61·06	23·00	0·952^*^
	Median	IQR	Median	IQR	
CRP (mg/l)	1·85	3·52	2·70	3·85	0·129^†^
ESR (mm)	18·00	21·00	15·00	10·00	0·281^†^
Complement C3 (mg/dl)	120·00	22·00	129·00	19·00	**0·010^†^**
Complement C4 (mg/dl)	22·00	10·00	27·00	11·00	**0·004 ^†^**

C reactive protein (CRP), Erythrocyte sedimentation rate (ESR), Body mass index (BMI), Low density lipoprotein (LDL), High density lipoprotein (HDL).

Bold values indicate statistically significant differences (p < 0.05).

* Student *t* test mean (sd) deviation.

† Mann Whitney *U* median (IQR).

As depicted in Table 3, the consumption of a pro-inflammatory diet was associated with higher body weight, BMI, body fat mass, hip and waist circumference, total cholesterol, triglycerides, LDL, and levels of complements C3 and C4 in crude analyses. Anthropometric measurements were associated with age, physical activity level, and disease score, and blood lipids were associated with E-DII independent of age, BMI, physical activity level, disease score, and smoking. In addition, Model 2 showed that each increase in E-DII score was associated with a 1·03 (95 % CI: 1·00, 1·05; *P*= 0·021) and 1·08 (95 % CI: 1·01, 1·13; *P*= 0·019) fold increase in complements C3 and C4, respectively.

**Table 3.** Logistic regression association of the E-DII with anthropometric measurements, blood lipids, and inflammatory markers (Odds ratios and 95 % confidence intervals)

	Crude			Model 1			Model 2		
Anthropometric measurements	OR	95 % CI	*P*	OR	95 % CI	*P*	OR	95 % CI	*P*
Body weight (kg)	1·05	1·01, 1·09	**0·013**	1·05	1·01, 1·08	**0·018**	1·04	1·01, 1·08	**0·019**
BMI (kg/m^2^)	1·12	1·03, 1·21	**0·006**	1·11	1·03, 1·21	**0·007**	1·11	1·03, 1·20	**0·009**
Lean body mass (kg)	1·00	0·95, 1·05	0·995	1·00	0·95, 1·05	0·996	0·99	0·95, 1·05	0820
Body fat mass (kg)	1·08	1·02, 1·14	**0·009**	1·10	1·02, 1·14	**0·008**	1·10	1·02, 1·14	**0·008**
Hip circumference (cm)	1·05	1·01, 1·09	**0·013**	1·05	1·01, 1·10	**0·014**	1·05	1·01, 1·10	**0·016**
Waist circumference (cm)	1·06	1·02, 1·10	**0·001**	1·06	1·02, 1·10	**0·002**	1·06	1·02, 1·10	**0·002**
Blood lipids									
Total cholesterol (mg/dl)	1·01	1·00, 1·02	**0·042**	1·01	1·00, 1·03	**0·030**	1·01	1·00, 1·03	**0·030**
TAG (g/dl)	1·01	1·00, 1·02	**0·019**	1·01	1·00, 1·02	**0·017**	1·01	1·00, 1·02	**0·018**
LDL-cholesterol (mg/dl)	1·01	1·00, 1·03	**0·042**	1·01	1·00, 1·02	**0·018**	1·02	1·00, 1·03	**0·021**
HDL-cholesterol (mg/dl)	1·00	0·98, 1·02	0·952	1·00	0·98, 1·01	0·763	1·00	0·98, 1·02	0·835
Inflammatory markers									
ESH	1·02	0·99, 1·05	0·225	1·01	0·98, 1·05	0·287	1·02	0·99, 1·06	0·168
CRP	1·00	0·99, 1·01	0·766	1·00	0·99, 1·01	0·743	1·00	0·99, 1·01	0·753
Complement C3	1·02	1·00, 1·04	**0·043**	1·03	1·00, 1·05	**0·023**	1·03	1·00, 1·05	**0·021**
Complement C4	1·07	1·00, 1·13	**0·015**	1·06	1·01, 1·13	**0·008**	1·08	1·01, 1·13	**0·019**

Anthropometric measurement; Model 1: age, physical activity level Model 2: age, physical activity level, disease score, drug use for SS.

Blood lipids; Model 1: age, physical activity level Model 2: age, BMI, physical activity level, disease score, drug use for SS, smoking.

Inflammatory markers; Model 1: age, disease score, smoking Model 2: age, disease score, drug use for SS, smoking, physical activity level.

Bold values indicate statistically significant differences (p < 0.05).

The distribution of daily energy and macro- and micronutrient intakes in the anti-inflammatory and pro-inflammatory diet groups according to the median value of E-DII scores of patients with PSS is presented in Table 4. The findings showed that the energy intake, total fat, saturated fat, and monounsaturated fat (MUFA) intake of individuals consuming the anti-inflammatory diet were statistically significantly lower than the values for those consuming the pro-inflammatory diet. However, no relationship was found between other macro- and micronutrients.

**Table 4.** Distribution of daily energy and macro- and micronutrient intakes according to the E-DII score (Mean values and standard deviations)

	Median E-DII ≤ –2·36 (*n* 51)		Median E-DII > −2·36 (*n* 51)		*P*
	Mean	sd	Mean	sd	
Energy, kcal	1515·53	322·32	1702·71	322·76	**0·004**
Carbohydrate, g	158·99	56·45	178·64	44·69	0·054
Protein, g	57·53	21·46	58·37	18·32	0·831
Fat, g	69·56	21·04	81·60	24·47	**0·009**
Cholesterol, mg	322·95	209·32	322·71	139·96	0·995
Saturated fat, g	25·26	8·97	31·45	12·66	**0·005**
MUFA, g	26·80	9·07	31·65	12·00	**0·023**
PUFA, g	11·31	8·30	12·18	6·26	0·552
Posa, g	21·45	8·40	21·68	9·80	0·900
Vitamin A, RE	863·61	3572·36	734·51	2418·21	0·831
Carotene, mcg	5794·41	7785·67	3942·75	4114·90	0·137
Vitamin D, IU	3·61	5·50	3·61	6·02	0·995
Vitamin E, mg	12·70	8·6	12·05	5·94	0·658
Thiamine, mg	0·90	0·35	0·88	0·29	0·702
Riboflavin, mg	1·65	0·85	1·49	0·63	0·270
Niacin, mg	11·69	6·30	12·21	8·74	0·733
Vitamin B_6_, g	1·24	0·54	1·20	0·50	0·646
Folate, mcg	363·88	182·90	323·31	147·31	0·220
Vitamin B_12_, mcg	5·81	14·62	3·98	7·34	0·428
Vitamin C, mg	147·13	96·22	120·27	90·01	0·149
Mg, mg	262·43	89·51	276·84	108·00	0·465
Fe, mg	9·35	3·35	10·29	4·27	0·221
Zn, mg	8·24	3·21	8·35	2·67	0·844
Se, mcg	67·90	29·89	60·92	37·18	0·298

MUFA monounsaturated fatty acid, PUFA polyunsaturated fatty acid, RE retinol equivalent.

Bold values indicate statistically significant differences (p < 0.05).

## Discussion

This study investigated the relationship between the inflammatory index of the diet and anthropometric measurements, disease activity, inflammatory markers, and blood lipids in 102 female patients with PSS. The study results showed a statistically significant association between E-DII and anthropometric measurements, inflammatory markers, and blood lipids in patients with PSS. However, no association was found between systemic disease activity as assessed by ESSDAI and E-DII.

Increased adipose tissue may cause chronic low-grade inflammation by increasing the secretion of inflammatory adipocytes^(19)^. In addition, high energy intake, simple carbohydrate intake, fat intake, and consumption of packaged food, fast food, and sugary drinks, compared with low intake of vegetables, fruits, and foods with high fiber content, can trigger obesity and inflammation^(20–22)^. Many studies have shown a relationship between DII and obesity. BMI, waist circumference, and fat mass of individuals increased with the increase in the pro-inflammatory properties of the diet^(23–27)^. Similar results were obtained in rheumatologic inflammatory diseases such as systemic lupus erythematosus and rheumatoid arthritis^(12,28)^. In this study, E-DII was correlated with body weight, BMI, waist circumference, hip circumference, and body fat mass measured using the bioimpedance scale. When the results were adjusted for age, physical activity level, disease score, and smoking, an increase in the anti-inflammatory content of the diet was found to be associated with a decrease in body weight BMI, waist circumference, hip circumference, and body fat mass.

Inflammation plays an essential role in lipid profile disruption^(29)^. This view is supported by several studies showing that the inflammatory characteristics of the diet are associated with the early determinants of cardiovascular diseases such as hypertension and lipid profile disorders^(30,31)^. In a study of 266 overweight or obese Iranian women, the DII score was significantly associated with lower HDL and higher triglyceride levels^(32)^. Similarly, a more pro-inflammatory diet, reflected by higher E-DII scores, was associated with pro-atherogenic lipoprotein profiles in the Cork and Kerry Diabetes and Heart Disease Study^(33)^. In another study on 454 patients with coronary artery bypass graft, male patients in the third and fourth quartiles with high DII scores and pro-inflammatory characteristics had significantly higher total cholesterol, triglyceride, creatinine, and high-sensitivity CRP (hsCRP) concentrations and lower HDL concentrations compared with male patients in lower quartiles. In contrast, only lipoprotein(a) concentrations differed between quartiles in female patients^(34)^. The cardiovascular disease risk and risk factors are seen more frequently in diseases with severe and intense inflammation^(35)^. Rheumatologic inflammatory diseases are also included in this group. In a study of 105 Spanish women with systemic lupus erythematosus, a positive correlation was found between DII and total cholesterol concentration, but no association was found with other cardiovascular risk factors such as HDL cholesterol, LDL cholesterol, triglycerides, hsCRP, and homocysteine concentrations^(36)^. In a study conducted on patients with rheumatoid arthritis, a significant positive correlation was found between DII score and elevated LDL cholesterol concentration, but no correlation was observed among total cholesterol, triglyceride, and HDL cholesterol concentrations^(13)^. The results of this study showed that patients with PSS with higher E-DII scores had higher total cholesterol, triglyceride, and LDL concentrations compared with those with PSS with lower E-DII scores when the E-DII score was separated from the median value. In addition, the logistic regression model indicated that this correlation was independent of age, disease score, smoking, BMI, and physical activity level.

The pro-inflammatory content of the diet affects inflammatory markers in diseases with lower inflammation compared with autoimmune or inflammatory diseases such as obesity, cardiovascular diseases, type 2 diabetes mellitus, and metabolic syndrome^(37)^. However, the results are not as evident as in other patients with severe and high inflammation. In two studies conducted on patients with systemic lupus erythematosus, the levels of inflammatory markers, such as tumour necrosis factor-*α* (TNF-*α*), CRP, and hsCRP, were not found to be associated with DII^(36–38)^. In a cross-sectional and longitudinal analysis using 6-year data from 208 patients with rheumatoid arthritis, the hsCRP levels were not associated with the DII score. In contrast, ESR was lower in those consuming anti-inflammatory diets^(39)^. In PSS patients, hypocomplementemia (C3 and C4) has been associated with disease activity and damage accumulation^(40)^. However, hypercomplementemia (C3 and C4) is also considered an indicator of inflammation^(41)^. Thus, in autoimmune diseases such as Sjogren’s syndrome, both hypocomplementemia and hypercomplementemia may pose risks. Complement proteins have been found to correlate positively with adipose tissue and obesity^(42)^. Furthermore, high-fat diets have been shown to induce complement activation, mediating intestinal inflammation and neoplasia independent of obesity^(41)^. Studies have demonstrated that low-calorie and weight-loss diets can improve complement C3 and C4 levels^(43,44)^. Similarly, diet-induced weight reduction has been reported to decrease C3 levels in individuals with abdominal obesity, potentially exerting a protective effect against cardiovascular diseases^(45)^. In this study, hypocomplementemia was not observed in either group. The present study found that the levels of complements C3 and C4 were significantly lower in patients with PSS who consumed anti-inflammatory diets than in those who did not. Similar results were obtained when these data were adjusted for age, disease score, smoking, BMI, and physical activity level. However, each unit increase in E-DII score had a low and limited effect on the levels of complements C3 and C4, with a 1·03 (95 % CI: 1·00, 1·05; *P*= 0·021) and 1·08 (95 % CI: 1·01, 1·13; *P*= 0·019) fold increase, respectively. The other inflammatory markers, including ESR and CRP levels, were not correlated with E-DII. It was unlikely that diet alone would have a large enough effect to change the levels of biomarkers easily, especially because patients with PSS actively experience inflammatory processes characteristic of the disease^(5)^. In addition, when the E-DII scores of the individuals participating in the study were compared with the previously reported scores, the anti-inflammatory content of the diets of patients with PSS (–4·01 to 1·16) was higher. Hence, the difference between the groups was not clearly reflected in inflammatory markers and disease activity^(25,46)^.

A statistically significant difference was noted in daily energy, total fat, saturated fat, and monounsaturated fat intakes between the anti-inflammatory and pro-inflammatory diet groups determined according to the median value of the E-DII score. However, no significant difference was found between the micronutrients with high anti-inflammatory and antioxidant properties. High-fat diet consumption and saturated fat intake have been shown to trigger intestinal dysbiosis, contributing to low-grade inflammation and decreased expression of anti-microbial peptides and gap junction proteins^(47)^. However, patients with PSS consuming a pro-inflammatory diet consume high amounts of not only total and saturated fatty acids but also monounsaturated fatty acids, which do not trigger inflammation due to the single double bond in their structure. The E-DII score recorded in this study was within a narrower range, influenced by the comparable intake of micronutrients with high anti-inflammatory and antioxidant properties in both groups, as well as the elevated MUFA content in their diets.

This study had some limitations, Sjögren’s disease represents a patient group with limited prevalence, making it challenging to achieve a larger sample size in this study. The energy intake of the patients was lower than that of the healthy population due to limited physical activity and the complication of dry mouth associated with the disease. The E-DII score was applied to address this. Although the patients who participated in the study were not given nutrition education before the study, the E-DII score was within a smaller range. This can be attributed to the prevalence of Mediterranean-style eating habits, which were more common among the patients due to the geographical region they inhabited.

In conclusion, this study showed that body weight, BMI, waist circumference, hip circumference, and body fat mass in patients with PSS were associated with E-DII score independent of age, physical activity level, disease score, and smoking. Similarly, total cholesterol, triglyceride, and LDL concentrations were associated with E-DII scores independent of age, physical activity level, disease score, BMI, and smoking. These associations were evident in anthropometric measurements and blood lipids but not in the case of inflammatory markers. No significant correlation was found between ESR and CRP levels and E-DII. In contrast, a significant correlation was found between E-DII and the levels of complements C3 and C4 when adjusted for age, disease score, smoking and physical activity level. Based on these findings, nutrition and the inflammatory content of the diet are considered essential factors in patients with PSS. However, further studies are warranted due to limited data and resource constraints in this field.

## Supporting information

Karataş et al. supplementary material Karataş et al. supplementary material

## Supplementary material

For supplementary material/s referred to in this article, please visit https://doi.org/10.1017/S0007114525103474
