# Supplementary material for: Association of the energy-adjusted dietary inflammatory index and Sjögren’s syndrome: a cross-sectional study
Source: Br J Nutr. 2025 Jun 3;133(11):1422–30. doi: 10.1017/S0007114525103474 (PMC12303722; doi:10.1017/S0007114525103474)
Supplement: Karataş et al. supplementary material [file S0007114525103474sup001.pdf]

KALİTE
Editing Services

CERTIFICATE OF EDITING

This document certifies that the manuscript listed below was edited for proper English language, grammar, punctuation, spelling, and overall style by one of the highly qualified native English speaking editors at Kalite Editing Services.

Manuscript title:

ASSOCIATION OF THE ENERGY-ADJUSTED DIETARY INFLAMMATORY INDEX WITH BLOOD LIPIDS, DISEASE ACTIVITY, AND INFLAMMATORY MARKERS IN PATIENTS WITH SJÖGREN'S SYNDROME: A CROSS-SECTIONAL STUDY

Authors:

Ezgi Karataş

Date issued:

January 2, 2024

Issued by:

Kalite Editing Services
Hasan FİLİK
General Manager

CERTIFIED
Editing

Disclaimer: Neither the research content nor the authors' intentions were altered in any way during the editing process. Documents receiving this certification should be English-ready for publication. The author is free to accept or reject our suggestions and changes in the edited document. However, we do not bear responsibility for revisions made to the document after our editing.

Certificate No: u2wv0uri

Bahcelievler Mah. 18. Cad. Aksa Sit. A-3 Blok Kat:5/32 Yenisehir/Mersin, Turkey
T:+90 324 327 17 11 F:+90 324 327 15 11
info@kaliteediting.com www.kaliteediting.com
